# Successful endoscopic hemostasis for waterfall‐like gastroduodenal artery hemorrhage

**DOI:** 10.1002/ccr3.7355

**Published:** 2023-05-26

**Authors:** Ryuhei Jinushi, Kazuya Koizumi, Sakue Masuda, Shomei Ryozawa

**Affiliations:** ^1^ Department of Gastroenterology Saitama Medical University International Medical Center Saitama Japan; ^2^ Department of Gastroenterology Medicine Center Shonan Kamakura General Hospital Kamakura Kanagawa Japan

**Keywords:** embolization, endoscopic, gastrointestinal hemorrhage, hemostasis, interventional, pancreatic neoplasms, radiology, therapeutic

## Abstract

Endoscopic hemostasis is the first step in cessation of gastrointestinal bleeding. Although IVR may sometimes be required for preventing rebleeding, prophylactic IVR was not considered necessary in this case because of complete endoscopic hemostasis.

A 75‐year‐old man under palliative care for pancreatic head carcinoma was admitted for anemia and fresh bloody stools. Table 1 shows the findings from the blood tests performed on admission. Esophagogastroduodenoscopy (EGD) on admission revealed an extensive deep‐seated ulcer in the descending duodenum suspicious of pancreatic cancer invasion with no findings suggestive of bleeding. Colonoscopy showed no abnormal findings. The patient was treated with omeprazole 40 mg/day intravenously and red blood cell transfusions. The following day, he passed fresh bloody stools and showed vitals of shock (blood pressure: 70/40 mmHg; pulse: 90/min). Repeat EGD showed a waterfall‐like spurting hemorrhage from the duodenal ulcerative lesion **(**Figure 1 **)**. Endoscopic hemostasis was performed using three reopenable clips **(**Figure 2 **)**. Interventional radiology (IVR) revealed that these clips were tightly ligated to the gastroduodenal artery **(**Figure 3 **)**. The patient's anemia improved, and he was discharged. In general, upper gastrointestinal bleeding is first observed endoscopically, and if necessary, endoscopic hemostasis is performed. However, in cases of arterial bleeding or large exposed blood vessels, preventive transarterial embolization (P‐TAE) may be performed to prevent rebleeding post‐endoscopic hemostasis.^1^, ^2^ In this case, the patient had a major hemorrhage from the gastroduodenal artery, but P‐TAE was unnecessary because endoscopic hemostasis was effective.

**TABLE 1:** Laboratory data.

Blood biochemistry			Complete blood cell count		
T‐Bil	0.7	mg/dL	WBC	12,900	/μL
AST	15	U/L	NEUT	88.9	%
ALT	11	U/L	LYM	6.1	%
LDH	148	U/L	MONO	4.7	%
γ‐GTP	38	U/L	EO	0.1	%
ALP	117	U/L	BASO	0.2	%
TP	5.7	g/dL	RBC	273 × 10^4^	/μL
Alb	2.8	g/dL	Hb	7.5	g/dL
BUN	11.8	mg/dL	Ht	23	%
Cre	0.6	mg/dL	MCV	84.2	fL
UA	4.5	mg/dL	Plt	36 × 10^4^	/μL
CK	27	U/L	Ret	1.6	%
CRP	7.0	mg/dL	Coagulation fibrinolysis examination		
Na	129	mmol/L	PT‐INR	1.2	
K	5.8	mmol/L	APTT	31.7	s
Cl	92	mmol/L	Fib	454	mg/dL
Ca	8.7	mg/dL	Infectious disease examination		
BS	161	mg/dL	HBs‐Ag	−	
Fe	17	μg/dL	HCV‐Ab	−	
TIBC	192	μg/dL	TP‐Ab	−	
Ferritin	113	ng/mL	RPR test	−	

Abbreviations: Alb, albumin; ALP, alkaline phosphatase; ALT, alanine aminotransferase; APTT, activated partial thromboplastin time; AST, aspartate aminotransferase; BASO, basophil; BS, blood sugar; BUN, blood urea nitrogen; Ca, calcium; CK, creatine kinase; Cl, chlorine; Cre, creatinine; CRP, c‐reactive protein; EO, eosinophil; Fe, ferrum; Fib, fibrinogen; Hb, hemoglobin; HBs‐Ag, hepatitis B surface antigen; HCV‐Ab, hepatitis C virus antibody; K, kalium; LDH, lactate dehydrogenase; LYM, lymphocyte; MCV, mean corpuscular volume; MONO, monocyte; Na, natrium; NEUT, neutrophil; Plt, platelet; PT‐INR, prothrombin time‐international normalized ratio; RBC, red blood cell; Ret, reticulocyte; RPR, rapid plasma regain test; T‐Bil, total‐bilirubin; TIBC, total iron binding capacity; TP, total protein; TP‐Ab, treponema pallidum antibody; UA, uric acid; WBC, white blood cell; γ‐GTP, γ‐glutamyl transpeptidase.

**FIGURE 1:**
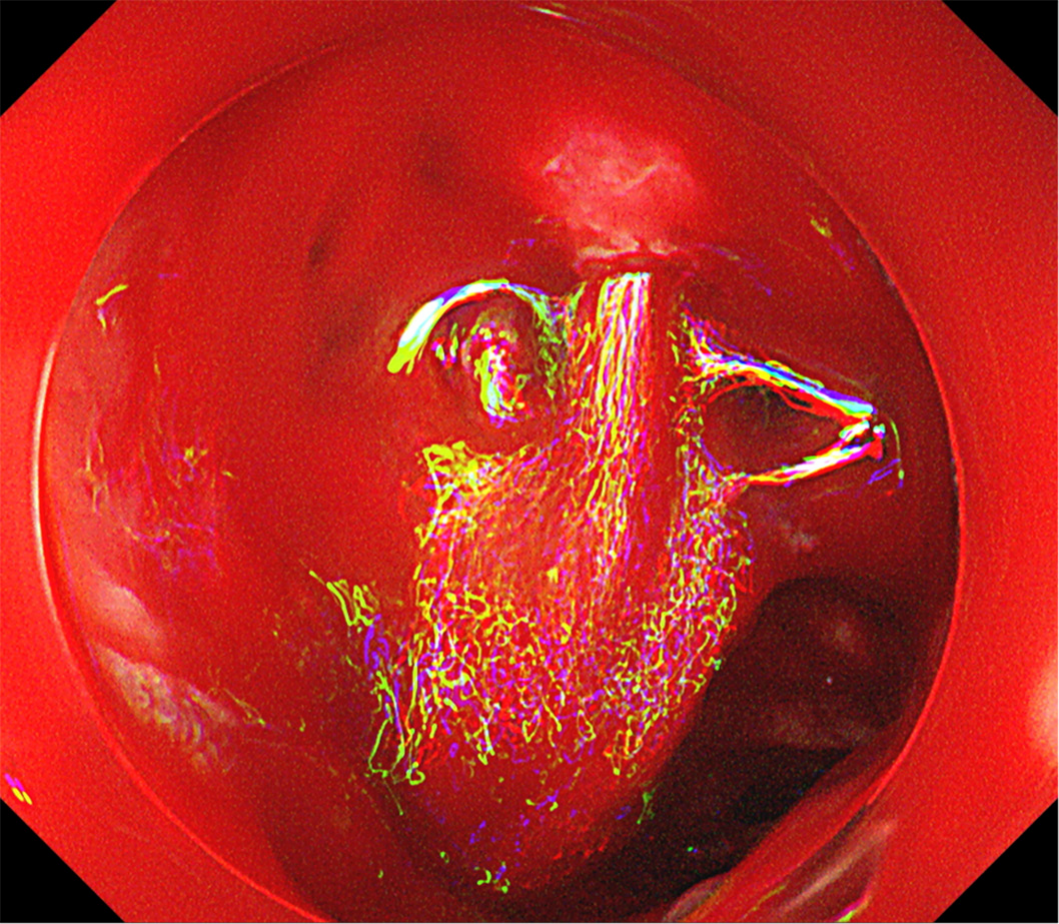
Endoscopic image. Spurting hemorrhage from the duodenal ulcerative lesion.

**FIGURE 2:**
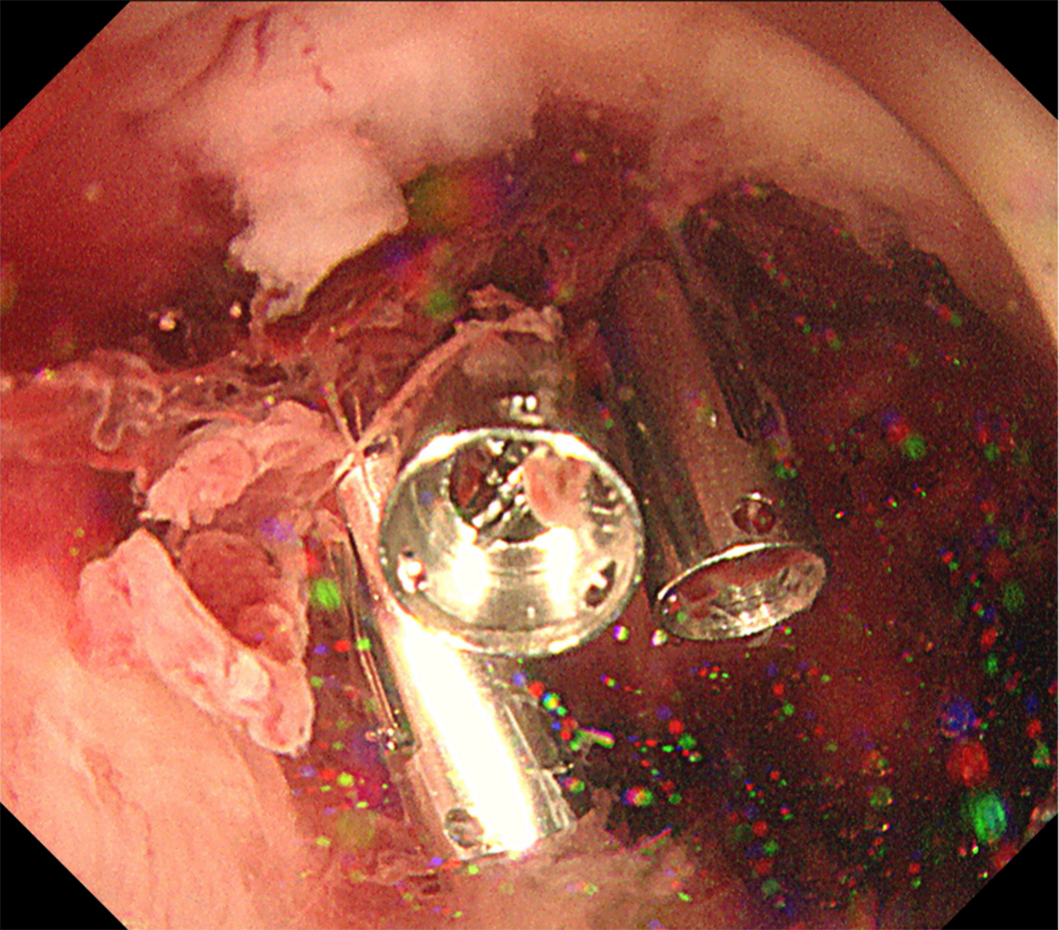
Endoscopic hemostasis. Complete endoscopic hemostasis achieved using three reopenable clips.

**FIGURE 3:**
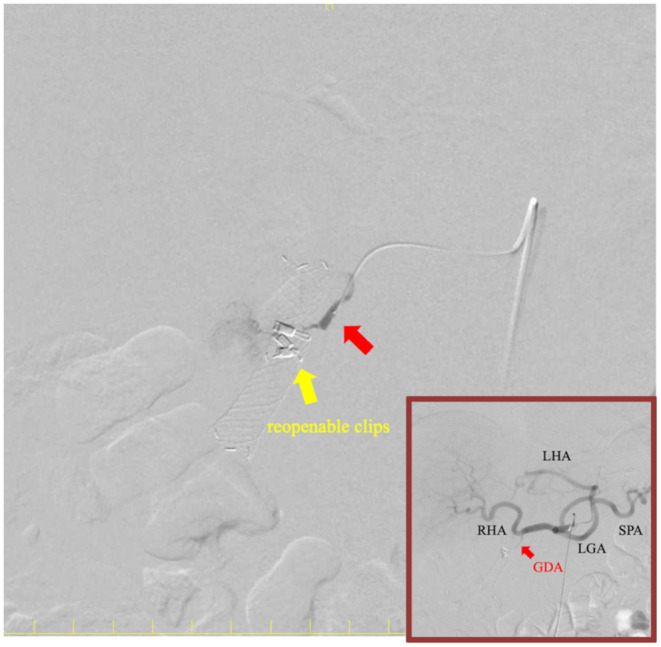
Prophylactic interventional radiology. Gastroduodenal artery (GDA) tightly ligated during primary endoscopic hemostasis. The left hepatic artery (LHA) branched off the left gastric artery (LGA). GDA; gastroduodenal artery; LGA, left gastric artery; LHA, left hepatic artery; RHA, right hepatic artery; SPA, splenic artery.

In future, it is necessary to determine whether endoscopic hemostasis alone is sufficient in cases of gastrointestinal bleeding, without the need for P‐TAE.

## AUTHOR CONTRIBUTIONS

**Ryuhei Jinushi:** Conceptualization; data curation; writing – original draft; writing – review and editing. **Kazuya Koizumi:** Writing – review and editing. **Sakue Masuda:** Writing – review and editing. **Shomei Ryozawa:** Writing – review and editing.

## FUNDING INFORMATION

None to report.

## CONFLICT OF INTEREST STATEMENT

The authors declare no conflicts of interest.

## CONSENT

Written informed consent was obtained from the patient for the publication of this report following the journal's patient consent policy.

## GUARANTOR

Ryuhei Jinushi, MD.

## Data Availability

The data supporting the findings of this study are available from the corresponding authors (RJ) upon reasonable request.
